# Evaluating the impact of universal Lynch syndrome screening in a publicly funded healthcare system

**DOI:** 10.1002/cam4.3279

**Published:** 2020-07-23

**Authors:** Petra W. C. Lee, Angela C. Bedard, Setareh Samimi, Vivienne K. Beard, Quan Hong, James E. J. Bedard, Blake Gilks, David F. Schaeffer, Robert Wolber, Janice S. Kwon, Howard J. Lim, Sophie Sun, Kasmintan A. Schrader

**Affiliations:** ^1^ Department of Biology University of the Fraser Valley Abbotsford BC Canada; ^2^ BC Cancer Hereditary Cancer Program Vancouver BC Canada; ^3^ Hematologie et Oncologie Departement Hôpital du Sacré‐Coeur de Montréal Montreal QC Canada; ^4^ Department of Pathology Vancouver General Hospital The University of British Columbia Vancouver BC Canada; ^5^ Division of Gynecology Oncology BC Cancer Vancouver BC Canada; ^6^ Department of Medical Oncology BC Cancer Vancouver BC Canada; ^7^ Department of Medical Genetics The University of British Columbia Vancouver BC Canada; ^8^ Department of Molecular Oncology BC Cancer Vancouver BC Canada

**Keywords:** colorectal cancer, endometrial cancer, DNA mismatch repair, genetic testing, immunohistochemistry, Lynch syndrome

## Abstract

**Purpose:**

Referrals for Lynch syndrome (LS) assessment have traditionally been based on personal and family medical history. The introduction of universal screening practices has allowed for referrals based on immunohistochemistry tests for mismatch repair (MMR) protein expression. This study aims to characterize the effect of universal screening in a publicly funded healthcare system with comparison to patients referred by traditional criteria, from January 2012 to March 2017.

**Methods:**

Patient files from the time of initiation of universal screening from 2012 to 2017 were reviewed. Patients were sorted into two groups: (a) universally screened and (b) referred by traditional methods. Mutation detection rates, analysis of traditional testing criteria met, and cascade carrier testing were evaluated.

**Results:**

The mutation detection rate of the universal screening group was higher than the traditionally referred group (45/228 (19.7%) vs 50/390 (12.5%), *P* = .05), though each were able to identify unique patients. An analysis of testing criteria met by each patient showed that half of referred patients from the universal screening group could not meet any traditional testing criteria.

**Conclusion:**

The implementation of universal screening in a publicly funded system will increase efficiency in detecting patients with LS. The resources available for genetic testing and counseling may be more limited in public systems, thus inclusion of secondary screening with *BRAF* and *MLH1* promoter hypermethylation testing is key to further optimizing efficiency.

## Graphical abstract

Lynch syndrome detection rates were higher in a universal screening cohort as compared to those referred by traditional phenotypic‐based referral criteria. Optimizing efficiency for Lynch syndrome screening is important in publicly funded systems where genetic counseling and other resources may be limited.

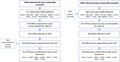

## 1 INTRODUCTION

Lynch syndrome (LS) is a hereditary cancer syndrome, characterized by increased risk for colorectal (CRC) and endometrial cancer (EC), among other cancers.^1^, ^2^ Mutations in the mismatch repair (MMR) genes *MLH1*, *MSH2*, *MSH6*, or *PMS2,* as well as *EPCAM*, are responsible for causing LS.^2^ Investigation for Lynch syndrome has traditionally been prompted by criteria met based on personal and/or family medical history or criteria such as Amsterdam I/II.^1^, ^3^ However, critics of the criteria argue they may be too exclusive and many individuals with LS may not be not referred for genetic testing.^4^

Universal screening is a newer process which uses immunohistochemistry (IHC) to test Lynch‐associated tumors for the presence of the four MMR proteins.^5^, ^6^ Patients with absent protein expression are referred for further testing.^6^ Universal screening is regarded as a more inclusive referral method compared to traditional methods, and is more consistent when used in the place of family health history.^7^, ^8^ A concern with universal screening is the cost of testing every tumor, but studies show that the process is relatively cost effective and quite beneficial.^1^, ^5^, ^8^, ^9^ MMR deficiency offers predictive utility with respect to use of 5FU monotherapy and immunotherapy with PD‐1/PDL‐1 antibodies.^10^, ^11^ Additionally, new sequencing techniques continue to be developed which will decrease the cost and time of universal screening.^1^

In 2012, the Vancouver Coastal Health Authority, which services a population of over 1 million residents, in the Canadian province of British Columbia implemented a universal screening program for all new CRC and EC diagnoses.^6^ In our province, the provision of health services is divided geographically into regional governance structures known as Health Authorities, which are responsible for both the delivery and administration of health care in a defined geographic area. To minimize costs, cases are screened for loss of MSH6 and PMS2 with reflex testing of the partner protein if one a protein is found to be absent, given that functionally, the MLH1 protein partners with PMS2 and the MSH2 protein partners with MSH6. CRC cases also are screened for presence of the *BRAF* V600E somatic mutation. Patients warranting further investigation and genetic counseling are referred to the provincial Hereditary Cancer Program (HCP).

Currently, there are few studies which assess the effect of universal screening in Canada. Universal screening has not replaced traditional referrals as standard practice among most health institutions; therefore, limited data are available.^12^ Our study aims to assess the impact of implementation of universal screening on the detection of individuals with Lynch syndrome in a publicly funded system. We focus on the comparison of universal screening to the traditional referral process.

## 2 MATERIALS AND METHODS

Approximately 750 patient files were compiled by the HCP’s clinical database manager to create the dataset. To be included, patients must have been referred for a LS assessment between January 2012 and March 2017. This timeline corresponds with the Vancouver Coastal Health Authority's implementation of universal screening to the project start date. The Health Authority contains six hospitals. Patients were sorted into two categories: referred by universal screening or referred by traditional methods. Universally screened patients must have had IHC testing on their tumor and a pathology report, which indicated that a referral to the HCP is suggested based on mismatch repair deficiency on IHC. The number of cases that underwent universal screening was acquired from the Health Authority, which maintained a separate research database for those cases. Cases which were not universally screened were considered referred by traditional methods.

The number of traditional testing criteria, according to HCP provincial guidelines, was tabulated for each patient. Individuals with a personal history of a Lynch syndrome‐related cancer were eligible for testing (“traditional testing”) if they met one of the following program testing criteria: the family meets the Amsterdam I or II criteria, the patient has 2(+) Lynch‐associated cancers with one at an age ≤50, two or more first‐degree relatives have a Lynch‐associated cancer, there are 3(+) cases of a Lynch‐associated cancer over more than one generation, the patient has an isolated case of CRC at an age ≤50, the patient is adopted with a personal history of CRC at an age ≤50, the patient has an IHC‐deficient or MSI‐high tumor (which was ordered on an individual basis, and not completed as part of a universal screening approach), other, or approved at clinical lab. The mean, median, and mode for criteria met were calculated.

Assessment of HCP testing criteria met required personal and familial health history of the patient. Questions were answered based on information provided by the index patient during genetic counselling appointments. When the exact age of diagnosis was not provided, estimates were made as follows: “early thirties” was 31, “mid‐thirties” was 35, and “late thirties” was 39. Absent MMR proteins and the tumor type tested for each patient were identified in pathology reports. Genetic reports for each patient who was diagnosed with LS were used to determine the type of germline mutation.

A cascade carrier testing analysis was completed for patients who were diagnosed with LS. The total number of eligible first‐degree relatives were counted for each index patient. To be counted as eligible, the relative had to be over the age of 18 and a presumed resident of British Columbia, if not stated otherwise. The number of relatives seen for a genetic counseling appointment and the number of carriers identified were also calculated. Test results for relatives were obtained from our provincial program database; the provincial program manages carrier testing for the entire province. Relatives tested out of province could not be included in the analysis.

### 2.1 Statistical analysis

Descriptive statistics were presented for both categorical and continuous variables. For categorical variables such as gender and mutation type, the frequency and the proportion were summarized. For continuous variables the mean with standard deviation (SD), or the median with range were calculated. To compare two groups of universal screening and traditional methods, Chi‐square test was conducted to analyze the difference in the mutation detection rates. All statistical analyses were two‐tailed with a statistical significance of *P* ≤ .05. Analysis was performed in the R Environment for Statistical Computing, version 3.4.3.

## 3 RESULTS

### 3.1 Universal screening vs traditional screening

**Figure 1:**
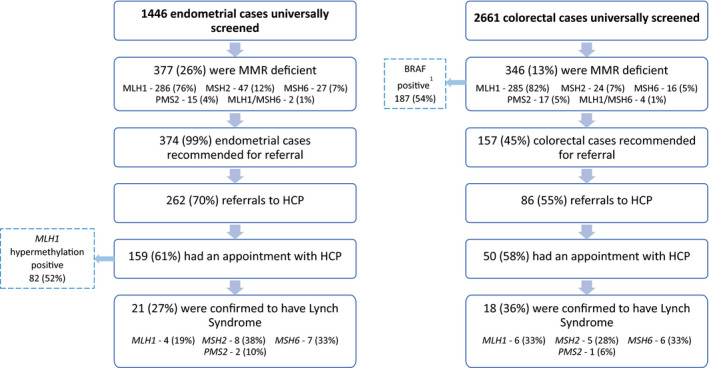
A, Results of the endometrial cancer universal screening process. B, Results of the endometrial cancer universal screening process. MMR, mismatch repair; HCP, hereditary cancer program. ^1^BRAF was completed for MLH1‐deficient cases only

Universal screening by the Vancouver Coastal Health Authority was performed for 2661 colorectal cancers, 1446 endometrial or ovarian (endometroid‐type) cancers, and 417 other cancers (Figure 1). Those recommended for referral were 157 colorectal cancers (26%), 374 endometrial/ovarian (endometrioid) cancers (63%) and 65 other cancers (11%). 358 cases followed through on referral to the HCP (60% overall referral rate); 86 colorectal cancers (55%), 262 endometrial (70%) and 34 other cancers (52%). Of the 358 referred, 228 had an appointment with the program during the study period of 2012‐2017. Of the 130 patients not seen during the study period, 51% were still on a waitlist, 24% could not be reached, 16% did not wish to have an appointment, and 9% were deceased. The universal screening cohort contained 28 males (12.3%) and 200 females (87.7%). The IHC test results show that 61.8% of referred cases were MLH1 deficient, and loss of expression of MSH2 made up 21.9% of the cases. The number of patients who met each of the traditional criteria can be found in Table 1. The mean number of traditional referral criteria that each patient met was found to be 1.4.

**Table 1:** HCP traditional testing criteria met by universally screened patients

Criterion	# of universally screened patients (n = 228)	# of LS positive universally screened patients (n = 45)	# of LS positive traditionally referred patients (n = 50)
Amsterdam I or II	20	14	25
Two or more cases of Lynch associated cancer (1 is CRC, 1 is ≤ 50)	9	4	7
Two or more first degree relatives with a Lynch associated cancer	4	2	1
Three cases of a Lynch associated cancer over more than one generation	35	19	28
Isolated case of CRC ≤50	8	1	1
Adopted and CRC ≤50	0	0	0
IHC‐deficient/MSI high	228	45	16
Other	18	4	2
Approved at clinical lab	3	1	6

Abbreviations: CRC, colorectal cancer; HCP, Hereditary Cancer Program; IHC, immunohistochemistry; LS, Lynch syndrome; MSI, microsatellite instability.

**Figure 2:**
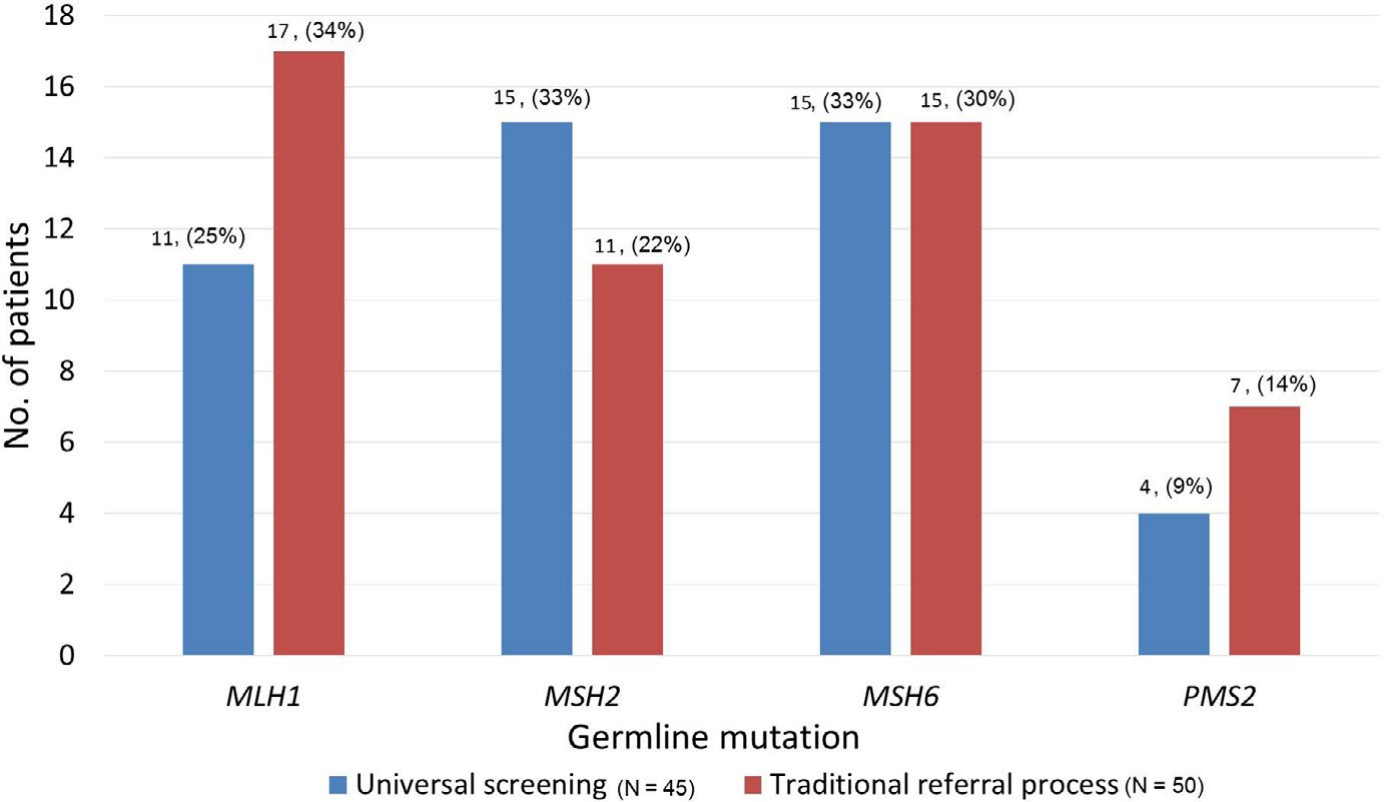
The number of germline mutations identified per gene by universal screening vs the traditional referral process

Of the 228 cases seen by HCP who were referred via universal screening, 45 were found to carry a germline mutation that causes LS. This gives an overall mutation detection rate of 19.7% for universal screening. The LS positive cases were composed of 13 males (28.9%) and 32 females (71.1%). Of the 45 patients diagnosed with LS, 11 had a mutation in the *MLH1* gene, 15 had a *MSH2* mutation, 15 had a mutation in the *MSH6* gene, and four had a *PMS2* mutation (Figure 2). The number of traditional criteria met by mutation‐positive patients referred through universal screening can be found in Table 1. The mean number of criteria that each patient met was calculated to be 1.98. *T* test was performed on the mean number of criteria patients met. The result revealed that patients with a positive mutation met significantly more criteria than patients without a mutation, *t* = 7.7209, *P* < .01.

There were 390 cases referred by traditional methods which resulted in the identification of 50 patients with LS and an overall detection rate of 12.8%. A germline mutation in *MLH1* was found in 17 patients, 11 had a mutation in *MSH2*, 15 had a mutation in *MSH6*, and seven patients had a *PMS2* mutation (Figure 2). The number of patients that met each of the traditional criteria can be found in Table 1. The mean number of criteria that each patient met was determined to be 1.72.

The detection rate was higher in the universal screening group (19.7%) as compared to the traditional criteria group (12.8%). The difference was statistically significant at *P* = .05.

Over the course of the study period universal screening using IHC for just the Lynch syndrome associated proteins was also implemented to some degree for colorectal and endometrial cancers diagnosed at two major hospitals within the Vancouver Island Health Authority in 2015. Analysis of cases referred from each center from 2015 to the end of the study period identified 15 cases referred on the basis of MMR deficient tumors alone (seven colorectal and eight endometrial cases), two of which were found to have Lynch syndrome. We included these cases in the traditional group since the tumor screening was implemented in a staged manner, as compared to being universally applied; if we were to exclude these cases from the traditional detection group, it would result in the same percentage mutation detection rate of 12.8% (48/375).

### 3.2 Colorectal cancer vs endometrial cancer

There were 50 universally screened patients with IHC testing done on colorectal tumors, 18 of which were diagnosed with LS. 159 patients were referred and seen based on the IHC testing done on their endometrial tumors, LS was diagnosed in 21 of these cases. The majority of the referrals were from patients with MLH1 absent endometrial cancer (n = 188); follow‐up *MLH1* promoter hypermethylation was performed on cases where patients consented to further testing (n = 95), and for those patients with negative hypermethylation (n = 13), 23% were found to carry a germline mutation. The traditional referral process identified 30 patients with LS that had colorectal tumors. Fifteen patients with endometrial tumors were diagnosed with LS.

### 3.3 Cascade carrier testing

A total of 170 relatives were seen for cascade carrier testing during the study period. 110 (64.7%) of these were first‐degree relatives and 32 (18.8%) were second‐degree relatives (Figure 2). The cascade testing identified 61 family members who were carriers of LS and confirmed segregation in 15 families (Figure 2). Of the 170 relatives, 89 (52.4%) were not carriers of the family mutation, 11 (6.5%) chose to decline testing after meeting with a genetic counsellor, and nine (5.3%) were awaiting results or for the sample to be taken. The number of relatives seen differed little whether the original proband had been found through universal screening (n = 83) or through the traditional criteria (n = 87).

## 4 DISCUSSION

This study is the first to describe the outcome of universal screening for LS in a Canadian setting. There was a significantly higher mutation detection rate in the universal screening cohort than for the patients referred traditional methods. Our results agree with the findings from other jurisdictions.^1^, ^13^ A recent survey of Canadian pathologists and genetic counselors were supportive of implementation of universal screening, with the promise that it could identify more patients with Lynch syndrome and help to guide treatment decisions,^14^ indicating our results may support some of the hopes expressed by these healthcare providers.

Alternative methods for initial LS screening were evaluated, and limitations in these methods were seen. The Amsterdam I or II criteria was met by only 14 (31.1%) of the universally referred patients. Similar to Beck et al^15^ and Walsh,^4^ this excludes almost 70% of mutation‐positive patients. Of the 50 patients with LS identified through the traditional referral process, 25 (50%) met the Amsterdam I or II criteria. This is an improved mutation detection rate compared to the universal screening cohort, but it still does not include a large percentage of the patients. Overall, there were 95 patients between the two groups that were diagnosed with LS syndrome, 39 of them could meet the Amsterdam I or II criteria. This gives an overall mutation detection rate of 41.1% and supports the finding that the Amsterdam criteria may be too exclusive to be used in a clinical setting.^15^

There was no specific testing criterion that performed exceptionally well and could be met by 60% or more of the patients diagnosed with LS. The HCP provincial guideline of ‘3(+) cases of a Lynch‐associated cancer over more than one generation’ had the highest detection rate of all testing criteria based on personal and familial health history. It was met by 19 (42.2%) of the universally screened LS patients and 28 (56.0%) of the patients referred by traditional criteria. While it performed better than the Amsterdam criteria for both groups, this criterion still cannot identify many patients with LS.

Though universal screening had the higher detection rate, there were cases that could have been missed by sole use of that method. Three traditionally referred cases might not have been detected by universal screening because each patient had normal IHC results which indicated expression of all MMR proteins. These patients met the Amsterdam II criteria and underwent germline testing which revealed a pathogenic mutation in each case. In one of the cases the IHC screen was done on a urothelial tumor block, since the prior colorectal tumor block was no longer available. Two other patients with normal IHC results, who were related to each other, were found to have an *MLH1* variant of uncertain significance on initial germline testing, but the variant was later reclassified to pathogenic based on protein function studies. There was one universally screened case that had IHC results which suggested that a *PMS2* mutation was present, but germline testing revealed it was a *MLH1* mutation. These four cases demonstrate that no method, including universal screening, has 100% accuracy and will be able to detect every single patient with LS. Overall, these data suggest that some integration of the two processes may be ideal for the clinical setting. This will include historic cases who were diagnosed when universal screening was not yet implemented, and for patients who live outside of the jurisdictions where universal screening is available.

The inclusion of secondary screening with *BRAF* and *MLH1* promoter hypermethylation testing is key to further optimizing efficiency. The number of endometrial tumors referred in the universal screening cohort was more than triple the number of colorectal tumors. This was not seen in the traditionally referred cohort. This is mainly due to the parallel screening of CRC cases for *BRAF* V600E, which explains somatic MLH1 loss of staining, which was not recommended for referral unless the personal or family history was otherwise concerning. A prescreen was not available for MLH1*‐*deficient endometrial cancer cases at the time of the study. The number of endometrial cases referred by universal screening could be significantly decreased by introducing *MLH1* methylation testing into the universal screening procedure. Only 3.2% (3/95) of the universal screen detected endometrial tumors with loss of MLH1 expression, who were seen for further genetics assessment, were found to have a germline mutation. Other studies have also shown the majority of MLH1‐deficient endometrial tumors to be hypermethylated.^16^, ^17^ The inclusion of hypermethylation analysis for *MLH1* in colorectal tumors would also represent an opportunity to screen out sporadic cases, as not all can be found by *BRAF* testing.^18^ Reflex hypermethylation testing would result in a fraction of the MLH1‐deficient tumors being referred for genetic testing, and a higher mutation detection rate, which would ultimately preserve limited genetic counseling and testing resources. As the adoption of universal screening by pathology centers becomes more widespread and may not be uniform in its ability to prescreen cases for likely sporadic cases using accompanying BRAF IHC or *MLH1* hypermethylation analysis, new modes of clinical triage that include brief medical history review and initiation of ancillary tests prior to appointment are being implemented to identify cases that may not require additional hereditary cancer assessment.

A challenge to the public health model for universal screening and long‐term cost effectiveness is when large numbers of patients are unable to access testing in a timely manner or choose not to undergo clarifying genetic investigations. Many referred patients were not yet seen for genetic counseling by the time of our analysis due to still being on our waitlist, but three other reasons were common, which may also relate to long waitlists: inability to reach the patient, patient decline of the appointment, or death. Long wait times for speciality care is a common concern for patients in Canada.^19^ As other publicly funded jurisdictions begin to introduce universal screening, this is a factor that needs to be considered. The patient decline rate for scheduling an appointment was relatively low at 16%. Universal screening studies in the United States have shown variable rates of decline, ranging from a 20%‐50% decline of genetic counseling appointments.^17^, ^20^, ^21^, ^22^ However, it is also possible a number of patients in our population declined the referral for genetic services in the first place, which may in part explain the relatively high number of cases that were not referred (40%). Other studies have shown that specialist referral is a challenge.^22^, ^23^ Increasing knowledge of LS by referring healthcare providers may help alleviate patient confusion and worry in the context of an initial positive LS screen. Further research could include reviewing the number of referrals per specialist group and ensuring that all specialists are aware of criteria for Lynch assessments. Additional solutions have been implemented in our program since the time of this study to help support these patients; these have included prioritizing MMR universal screen positive patients to an expedited appointment (wait‐time <3 months), and identification and follow‐up by a nurse navigator for patients needing referral from the gynecology‐oncology specialists. These are in keeping with the study of Canadian healthcare providers expressing support of implementation of universal screening for LS, provided that patients can opt‐out of further investigation.^14^, ^24^

For successful implementation of universal screening, adequate genetic counseling resources are required.^24^ At the time of evaluation, over 65 patients were still on the waitlist for genetic counseling services. In a publicly funded system, genetic counselors may be employed at smaller numbers than can support expeditious appointments for referrals from a universal screening program. In the United States, it is estimated that one per 100 000 genetic counselors is needed to adequately serve the population.^25^ In our province, there are approximately one per 300 000‐400 000 genetic counselors specifically working in the hereditary cancer field. Parallel increases in the workforce size as well as moves toward alternate service delivery models in genetic counseling^26^ will be needed to meet the increasing demand brought by universal screening.

We found that there were 401 first‐degree relatives eligible for carrier testing. These relatives all have a 50% chance of carrying a mutation, but the majority have not been seen by the HCP. Only 27.4% of the eligible first‐degree relatives made an appointment for genetic counseling. Wang et al^27^ had similar results with approximately 34.0% family participation after a mutation was identified, but it is currently unclear what causes this low follow through. Of the 95 eligible families, there was not a single relative seen from 46 (48.4%) of the families. Tomiak et al^28^ found that the term “genetic counselling” was a deterrent for patients to book an appointment because they equated the process with psychological counselling. Programs may consider this when developing outreach and educational material for patients. The need for more robust rates of cascade carrier testing in hereditary cancer has recently been called for,^29^, ^30^ and requires renewed energy by programs to examine outreach and services for this highest risk population. The diagnosis of the index patient is only one portion of a hereditary cancer program's goal to reduce morbidity and mortality related to LS in a given jurisdiction. To reach this goal, the family outreach portion of the program needs significant improvement. A limitation in our cascade carrier analysis is the inability to track results for family members tested out of province, which indicates the cascade carrier rate is likely underestimated. However, to what degree it is underestimated we cannot be certain, given that the address of all first‐degree relatives is not routinely collected.

Expanding implementation of universal screening for LS in Canada will bring the opportunity of finding more individuals and families at high risk for cancer. However, health authorities should consider standard *MLH1* methylation testing of endometrial tumors. Our study showed that a large portion of the universal screening cohort did not require referrals due to sporadic *MLH1* methylation. This change could decrease genetic counseling wait times and increase the mutation detection rate of universal screening, furthermore it would free up resources to pursue more aggressive cascade carrier testing and case finding in mutation‐positive families. Further research and improvements to the referral methods will bring us closer to the goal of identifying all carriers of LS.

## CONFLICT OF INTEREST

None declared.

## AUTHOR CONTRIBUTIONS

Petra Lee: Conceptualization, data curation, formal analysis, methodology, writing ‐ original draft, writing ‐ review, and editing. Angela Bedard: Conceptualization, data curation, formal analysis, methodology, writing ‐ original draft, writing ‐ review, and editing. Setareh Samimi: Conceptualization, data curation, formal analysis, methodology, writing ‐ review, and editing. Vivienne Beard: Data curation, formal analysis, methodology, writing ‐ review, and editing. Quan Hong: Data curation, formal analysis, methodology, writing ‐ review, and editing. James Bedard: Conceptualization, methodology, writing ‐ review, and editing. Blake Gilks: Conceptualization, writing ‐ review and editing. David Schaeffer: Writing ‐ review and editing. Robert Wolber: Writing ‐ review and editing. Janice Kwon: Conceptualization, writing ‐ review and editing. Howard Lim: Conceptualization, writing ‐ review and editing. Sophie Sun: Conceptualization, writing ‐ review and editing. Kasmintan Schrader: Conceptualization, methodology, writing ‐ review, and editing.

## Data Availability

The data that support the findings of this study are available on request from the corresponding author. The data are not publicly available due to privacy or ethical restrictions.
